# Impact of Laparoscopic Fundoplication for the Treatment of Laryngopharyngeal Reflux: Review of the Literature

**DOI:** 10.1155/2012/291472

**Published:** 2011-12-15

**Authors:** Guilherme da Silva Mazzini, Richard Ricachenevsky Gurski

**Affiliations:** ^1^Gastrointestinal Surgery Department, Hospital de Clínicas de Porto Alegre, Ramiro Barcelos Street 2350, 90035-903 Porto Alegre, RS, Brazil; ^2^Biochemstry Department, ICBS, Universidade Federal do Rio Grande do Sul, Ramiro Barcelos Street 2600 anexo, 90035-000 Porto Alegre, RS, Brazil; ^3^Surgery Department, Medicine School, Universidade Federal do Rio Grande do Sul, Ramiro Barcelos Street 2400, 90035-003 Porto Alegre, RS, Brazil

## Abstract

Laryngopharyngeal reflux (LPR) is part of the so-called extraesophageal manifestations of gastroesophageal reflux disease (GERD). It is presented by unspecific symptoms and signs and is believed to be caused by the reflux of gastric content to the proximal esophagus and larynx. However, evidence considering the role of the antireflux surgery for LPR has failed to demonstrate results comparable to those for GERD. The aim of this paper is to review the current literature regarding the impact of laparoscopic fundoplication for the treatment of LPR.

## 1. Introduction

Gastroesophageal reflux disease (GERD) is defined as a condition that develops when the reflux of stomach contents into the esophagus causes troublesome symptoms and/or complications and represents the failure of the antireflux barrier [1]. It has long been recognized as a significant public health concern, since heartburn afflicts nearly two-thirds of US adults at some point in their lives and accounts for a great number of physician office visits every year [2]. While most GERD patients suffer from typical esophageal manifestations, such as heartburn and regurgitation, there is a subset of patients who experience the so-called laryngeal symptoms, which may be caused by laryngopharyngeal reflux (LPR) [3].  Table 1 displays the most frequent symptoms associated with LPR, which are unspecific and can be found in other otolaryngologic disorders [4].

Despite the advances in medical management for GERD, the surgical treatment has been studied more methodically since the introduction of laparoscopic Nissen fundoplication [5], and increasing numbers of patients have opted for the surgical treatment since the advent of this minimally invasive technique. However, most of the studies regarding the surgical treatment for LPR symptoms have failed to show results as good as those for GERD symptoms [6]. Interestingly, the efficacy of medical treatment for LPR is also not comparable to that for GERD [7].

Although LPR and GERD have different clinical presentation and response to management, accumulating evidence shows the correlation between the pathophysiology of both entities, which is the reflux of the gastric content to esophagus or proximally, to the larynx [8]. Perry et al. 2008 [9] showed that, in the upright position, LPR patients have the same degree of gastric cardia dilation that is found in patients with typical GERD symptoms and those with a mixed presentation, suggesting that the same pathophysiologic disturbance that predisposes typical GERD patients to reflux is present in patients with symptoms of LPR. Furthermore, acid reflux is thought to lead to not only laryngeal alterations associated with LPR, but also the direct contact of the laryngeal epithelium with gastric refluxate containing pepsin, bile acids, and other components, which are not prevented by the proton pump inhibitors (PPIs) [8]. Taking together, this evidence suggests that the antireflux surgery should have good results in LPR patients as well, since it provides an effective barrier to gastroesophageal reflux and eliminates both acid and nonacid reflux.

The aim of this paper is to present the current evidence regarding the impact of laparoscopic fundoplication (LF) for the treatment of LPR.

## 2. Materials and Methods

A Medline, PubMed, and Cochrane database search was done to find articles in the English language on surgery for LPR in adults. The following keywords were used: “surgery/fundoplication and extraesophageal manifestations of gastroesophageal reflux,” “surgery/fundoplication and laryngopharyngeal reflux,” and “surgery/fundoplication and atypical symptoms of gastroesophageal reflux.” Related articles and links were searched. Additional articles were identified by a manual search of the references from the key articles. only articles regarding antireflux surgery specifically for the treatment of LPR and not including lower respiratory or other extraesophageal symptoms of GERD were selected.

## 3. Results

A total of five studies were selected. There were no randomized clinical trials and only one study had a control group. The inclusion criteria, preoperative evaluation, and endpoints are not standardized, making the data too heterogeneous.

Westcott et al. 2004 [10] studied 41 patients submitted to LF due to LPR symptoms. After a mean follow-up time of 14 months, he observed 84% improvement in reflux symptom index (RSI). From the preoperative evaluation, the factors significantly associated with poor outcome after surgery were structural changes seen in laryngoscopy (i.e., vocal-cord scarring, paresis, granuloma, and carcinoma or subglottic stenosis) and no improvement with PPIs treatment.

Swoger et al. 2006 [11] conducted a controlled study with 25 patients presenting LPR symptoms that did not respond to aggressive PPI treatment (omeprazole 40 mg twice daily or lansoprazole 60 mg twice daily for 4 months). From this group, patients who decided to submit to LF (*n* = 10) were compared to the ones who decided to keep on medical management (control group *n* = 15). No significant improvement in symptom scale was observed in both groups, despite a significant improvement in pH and laryngoscopy scores after surgery. Some patients have demonstrated improvement in symptoms by treating additional pathologies such as allergy or asthma.

Catania et al. 2007 [12] studied 58 patients submitted to LF for laryngopharyngeal reflux, for a mean followup of 15.4 months. Since the first month after surgery, he observed 97% improvement in symptoms (decrease >5 points in RIS) and 65% of total response (patients experiencing no symptoms). These results were maintained on late follow-up evaluation. Also, there was a significant increase in quality-of- life index used measured by laryngopharyngeal reflux-health-related quality-of-life index.

Sala et al. 2008 [13] evaluated vocal and laryngeal symptoms in 22 patients submitted to LF for LPR, after a 3 months course of medical treatment. Vocal and laryngeal symptoms significantly improved after 3 months of medical treatment and kept improving after surgery, showing a statistically significant difference between pre-and postsurgical treatment too. However, voice quality and laryngeal findings only showed a significant improvement after surgery.

Wassenaar et al. 2011 [14] introduced a new approach to preoperative evaluation. While most of the studies use classical evaluation with dual-probe 24 h-esophageal pHmetry, upper endoscopy, laryngoscopy, in this study, patients were additionally evaluated with laryngeal pepsin measured by western blotting in sputum and posterior laryngeal biopsies. All but one patient with LPR symptoms were positive for pepsin in laryngeal biopsy before surgery. Also, sputum was collected preoperatively in 5 patients and 4 of these were positive for pepsin, in correlation with correspondent biopsy. Seven patients were submitted to LF, and 2 were submitted to endoscopic fundoplication (EsophyX, EndoGastric Solutions, Redmond, Wash). From these 2 patients, 1 had to be submitted to LF for failure of the endoscopic treatment. Eight patients had symptom improvement (6 good improvement and 2 mild), and 1 had no improvement. From the 8 patients who experienced improvement, 7 were negative for pepsin in postoperative sputum analyses, and 1 had a consistent decrease in pepsin (from +++ preoperatively to + after surgery). The only one patient who did not experience improvement was negative for pepsin in preoperative biopsies and sputum.

## 4. Discussion

Literature review demonstrated additional studies regarding surgical treatment for LPR, but including patients with other extraesophageal symptoms, such as lower respiratory symptoms [15, 16]. We decided to exclude those studies from this paper in order to achieve a more specific analysis, since even the studies directed to LPR present critically heterogeneous evidence. Whereas most studies had shown some degree of symptomatic improvement after surgical fundoplication, further conclusions are challenging, due to the weak evidence grade of the studies.

A possible explanation to the difficulty of the studies in proving the efficacy of LF for LPR is that, in most of the studies, the diagnosis of LPR is focused on traditional measures of gastroesophageal reflux (esophagoscopy or pH monitoring), identification of injury by laryngoscopy, pharyngeal pH monitoring, or empiric treatment of symptoms by PPIs, which have demonstrated not to be reliable diagnostic tools [6]. LPR presents with a spectrum of symptoms and signs that are very unspecific and most of the times not associated to classical GERD symptoms [17]. Therefore, any advance in preoperative evaluation that could lead to a more specific characterization of the LPR and the correlation of the symptoms with the gastric reflux will help to study the effect of the restoration of the antireflux barrier in those patients.

Recent studies have focused on more specific methods for diagnosis and prediction of response to treatment in LPR patients [18]. Although not controlled and with a small number of patients, the study by Wassenaar et al. 2011 [14] probably brought a more specific marker for LPR, with a good correlation between symptoms and reflux, which marked even the efficacy of LF. Pepsin in sputum and/or in laryngeal biopsies must now be studied in large randomized controlled trials.

Another important factor to be taken into account is the body mass index (BMI) of subjects submitted to surgery. From the studies presented above, only one has provided the BMI of the patients [14]. Several studies have proposed a causative role for obesity on GERD [19, 20], but the correlation between obesity and LPR is still poorly understood [21, 22]. Additionally, long-term control of GERD by LF in obese patients seems to be worse than in normal weight subjects [23]. Therefore, patients BMI must be well characterized in any study regarding the efficacy of LF.

## 5. Conclusion

Results of LF for the treatment of GERD are well established [24], and, in theory, both GERD and LPR share a similar pathophysiology. Consequently, well-indicated antireflux surgery should be as effective for LPR as for GERD. Many studies have demonstrated symptomatic improvement after surgical fundoplication. However, the current knowledge presented by the literature does not allow this conclusion. Large multicenter, randomized control trials are needed, focusing on diagnostic tools to improve selection criteria, presenting standard end-points and long-term followup.

## Figures and Tables

**Table 1:** Laryngopharyngeal reflux symptoms.

Dysphonia
Swallowing difficulty (pseudodysphagia)
Globus
Throat clearing
Cough
Choking
Post nasal drip
Laryngospasm
Sore throat
